# Editorial: Rising stars in: evolutionary psychology 2022

**DOI:** 10.3389/fpsyg.2024.1376132

**Published:** 2024-02-21

**Authors:** Katherine Aumer, Guy J. Curtis, Peter K. Jonason

**Affiliations:** ^1^Division of Social Sciences, University of Hawaii–West Oahu, Kapolei, HI, United States; ^2^School of Psychological Science, University of Western Australia, Perth, PA, Australia; ^3^Department of General Psychology, University of Padua, Padua, Italy

**Keywords:** evolutionary psychology, multidisciplinary collaboration, musicality, birth order, body perception, sustainability, parental investment

For the past several decades the field of evolutionary psychology has significantly contributed to our understanding of the evolved nature of human cognition, behavior, and emotion. To reveal the latest insights in the field, we presented the Research Topic: *Rising Stars in Evolutionary Psychology 2022*. In this editorial, we endeavor to integrate insights garnered from these rising stars, who elucidated distinct facets of human experience through the lens of evolutionary psychology. Our analysis navigates the intricate interplay of biological heritage, reproductive strategies, masculine perceptions, and environmental conscientiousness to demonstrate the need for continued methodological refinement and cross-disciplinary collaboration.

Varella posits a paradigmatic hypothesis concerning the evolutionary trajectory of human musicality. The theory centers on the expansion of nocturnal activities throughout human evolution, identifying the night-time environment as a crucial factor in the development of human musicality. Drawing from independent principles, the hypothesis offers a nuanced perspective on the multifaceted roles assumed by human musicality in response to ancestral adaptive challenges/opportunities.

The study implicates nocturnal adaptive challenges such as low luminosity, imminent danger, and concealment of identity as key factors that may have selected for traits conducive to acoustic communication and imaginative, gregarious, and risk-taking dispositions. The integration of musicality into survival and reproductive roles is discussed within the context of the night-time adaptive landscape, shedding light on the evolutionary underpinnings of various musical features and functions.

Corpuz et al. extend the exploration into evolutionary dynamics, specifically focusing on the tradeoff between current and future reproduction from a life history theory perspective. Departing from the conventional focus on female sexual developmental milestones, the article turns toward male sexual developmental milestones to provide a more inclusive model of parental investment. The study measured sexual maturation using three indicators: thorarche, age at sexual debut, and the difference between thorarche and reproduction to predict the time invested in direct parental care. Corpuz et al. utilized the Experience Sampling Method (ESM) to overcome biases and limitations imposed by self-report. The unexpected finding that males with earlier sexual debut invest more time in infant care challenges established paradigms and underscores the complexity of reproductive timing and parental investment.

This departure from the typical, albeit limited narrative of paternal investment models prompts a reconsideration of the relationship between sexual maturation and paternal investment. The study's implications invite a nuanced examination of fatherhood that encourages the use of diverse sampling methods and demographic considerations.

In the domain of masculinity, Christensen et al. employ meticulous scrutiny of self-reported bodily markers, specifically in the tendency of men, at the aggregate level, to overestimate certain bodily measures linked to masculinity: penis size. Furthermore, it introduces the intriguing influence of monetary rewards on data quality, presenting a novel dimension to the methodological considerations of such studies.

The cautionary note regarding the interpretation of self-reported measures of penis size urges the need for refinement in research methodology. The incorporation of monetary incentives as a factor influencing data quality adds a layer of complexity to the design of studies relying on self-reported bodily markers. Christensen et al., similar to Corpuz et al. both found the importance of extending beyond the self-report method when collecting information that is sensitive to societal expectations.

Speaking of societal expectations, the current focus on environmental conscientiousness and sustainability has increased recently. To discover more about the factors surrounding green consumption, Otterbring et al. introduced an unexplored dimension—the influence of birth order. The study, conducted on a sample of 335 participants, revealed a small-to-moderate effect size, indicating that firstborns exhibit lower concerns linked to environmental protection in their purchase patterns. This unexpected revelation prompts a reconsideration of birth order dynamics in relation to broader societal issues and environmental challenges.

The findings, while modest in effect size, present informative nuances that warrant consideration, especially given the ease with which birth-order data can be collected. The implications of birth order on environmental concerns may be applied in a variety of contexts including economics, marketing, and legislation.

In this eclectic group of studies, evolutionary psychology, life history theory, scrutinized masculinity, and birth order dynamics converge and extend beyond disciplinary boundaries. The unexpected findings and nuanced dimensions revealed by these articles beckon researchers to delve deeper, question assumptions, and refine methodologies. These rising stars have demonstrated the strength of data collection methods that extend beyond self-report and the power of integration of disciplines. The scientific pursuit of understanding the ever-evolving nature of human cognition, behavior, and emotion will benefit from this continued collaboration.

## Author contributions

KA: Conceptualization, Data curation, Formal analysis, Investigation, Methodology, Project administration, Validation, Writing—original draft, Writing—review & editing. GC: Writing—review & editing. PJ: Supervision, Writing—review & editing.

